# Supplementary material for: Online single-cell data integration through projecting heterogeneous datasets into a common cell-embedding space
Source: Nat Commun. 2022 Oct 17;13:6118. doi: 10.1038/s41467-022-33758-z (PMC9574176; doi:10.1038/s41467-022-33758-z)
Supplement: Supplementary file 1 — Supplementary information [file 41467_2022_33758_MOESM1_ESM.pdf]

Online single-cell data integration through projecting heterogeneous datasets into a common cell-embedding space

Lei Xiong[1,2,4,8], Kang Tian[1,2,8], Yuzhe Li[1,3], Weixi Ning[1], Xin Gao[5,6,7], Qiangfeng Cliff Zhang[1,2]

[1] MOE Key Laboratory of Bioinformatics, Beijing Advanced Innovation Center for Structural Biology & Frontier Research Center for Biological Structure, Center for Synthetic and Systems Biology, School of Life Sciences, Tsinghua University, Beijing 100084, China

[2] Tsinghua-Peking Center for Life Sciences, Beijing 100084, China

[3] Academy for Advanced Interdisciplinary Studies, Peking University, Beijing, China 100871

[4] Shanghai Qi Zhi Institute, Shanghai 200030, China

[5] Computer Science Program, Computer, Electrical and Mathematical Sciences and Engineering (CEMSE) Division, King Abdullah University of Science and Technology (KAUST), Thuwal, 23955-6900, Kingdom of Saudi Arabia

[6] KAUST Computational Bioscience Research Center (CBRC), King Abdullah University of Science and Technology (KAUST), Thuwal, 23955-6900, Kingdom of Saudi Arabia

[7] BioMap, Beijing 100086, China

[8] These authors contributed equally

Correspondence should be addressed to Q.C.Z. (email: qczhang@tsinghua.edu.cn).

This PDF file includes:

Supplementary Figures

Supplementary Fig. 1 | Three design elements to learn a generalized encoder.
Supplementary Fig. 2 | Comparisons of integration performance of indicated methods across the indicated benchmark datasets.
Supplementary Fig. 3 | Comparisons of clustering results of indicated methods across the indicated benchmark datasets.
Supplementary Fig. 4 | Comparisons of integration performance by quantification metrics.
Supplementary Fig. 5 | Comparisons of integration performance of indicated methods based on Human Fetal Atlas dataset.
Supplementary Fig. 6 | Comparisons of integration performance of indicated methods based on scATAC-seq dataset and other modality datasets.
Supplementary Fig. 7 | Comparisons of integration performance of indicated methods based on cross-modality dataset.
Supplementary Fig. 8 | Canonical marker genes of different cell-types and UMAP embeddings of the *liver* dataset.
Supplementary Fig. 9 | Comparisons of integration performance based on partially overlapping simulated *pancreas* dataset.
Supplementary Fig. 10 | Comparisons of integration performance based on partially overlapping simulated *PBMC* dataset.
Supplementary Fig. 11 | Projection of three additional pancreas data batches onto the *pancreas* dataset.
Supplementary Fig. 12 | Projection of two melanoma datasets onto the *PBMC* dataset.
Supplementary Fig. 13 | Comparisons of integration across Atlas-level datasets.
Supplementary Fig. 14 | The SCALEX Mouse Atlas.
Supplementary Fig. 15 | The SCALEX Human Atlas.
Supplementary Fig. 16 | COVID-19 immune landscape.
Supplementary Fig. 17 | COVID-19 heterogeneous dysfunctional immune response.
Supplementary Fig. 18 | Projection of the SC4 Atlas onto the SCALEX COVID-19 PBMC Atlas.
Supplementary Fig. 19 | Ablation studies of different SCALEX architectures for single-cell data integration.
Supplementary Fig. 20 | Ablations studies of different SCALEX architectures for single-cell data projection.
Supplementary Fig. 21 | Comparison of SCALEX with beta=0.5 and beta=1 across the indicated benchmark datasets.

Supplementary Fig. 1

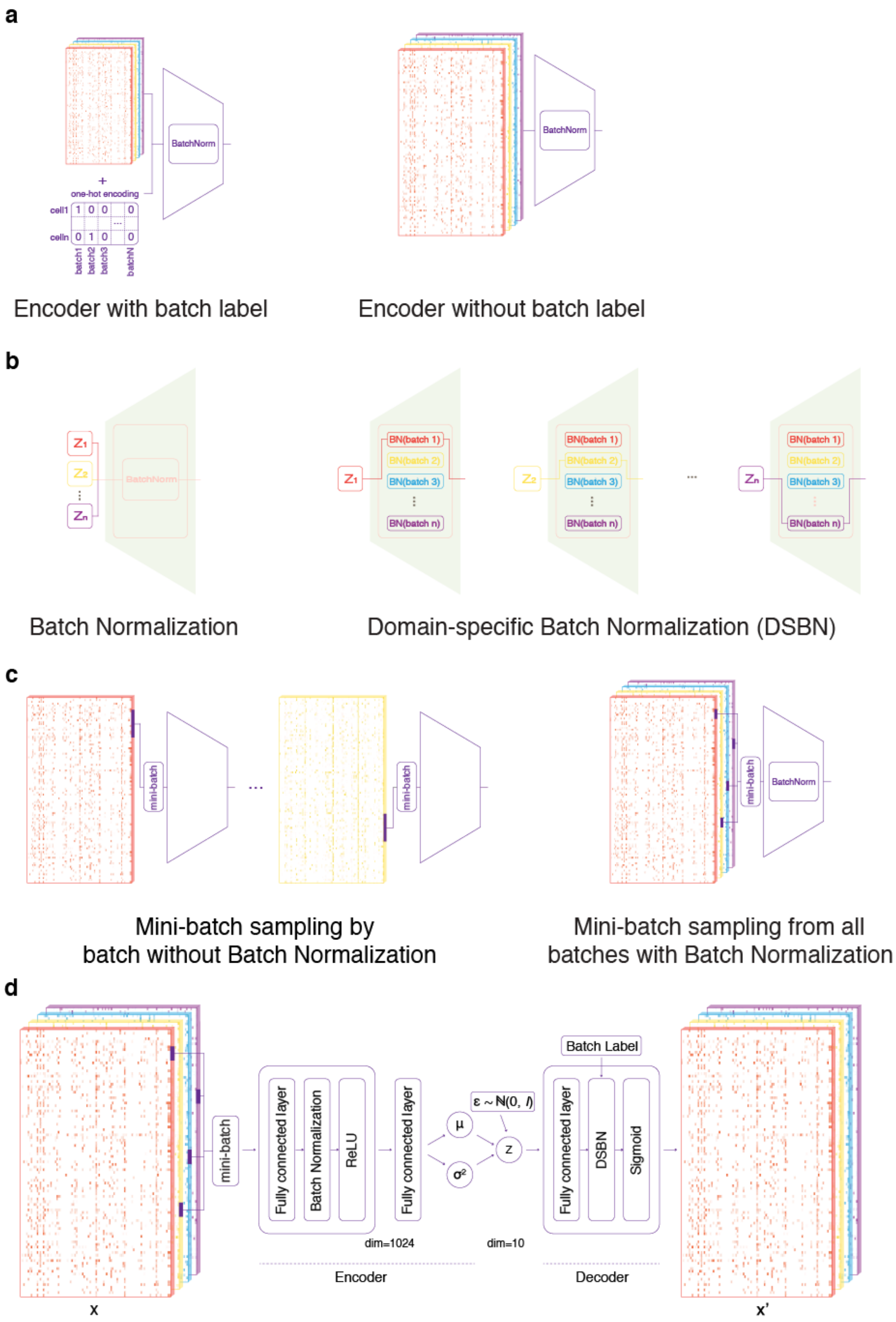

Supplementary Fig. 1 | Three design elements to learn a generalized encoder. a, Assessing encoders with or without batch labels. **b**, Assessing decoders with or without DSBN. **c**, Assessing mini-batch sampling by batch without Batch Normalization or sampling from all batches with Batch Normalization. **d**, Detailed architecture of SCALEX and information flow.

Supplementary Fig. 2

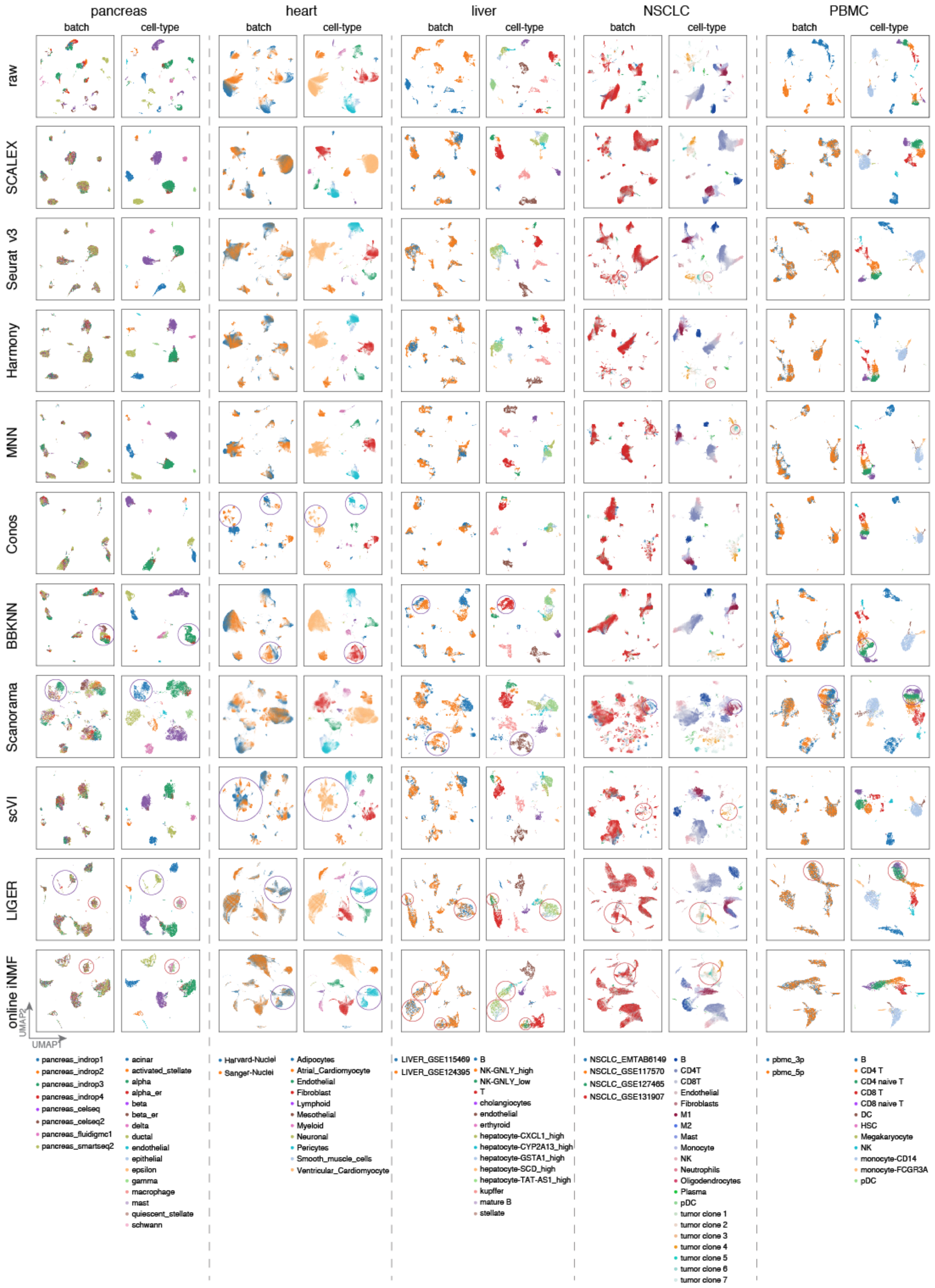

Supplementary Fig. 2 | Comparisons of integration performance of indicated methods across the indicated benchmark datasets. UMAP embeddings of the indicated methods across the indicated benchmark datasets colored by batches and cell-type. Misalignments are highlighted with red circles.

Supplementary Fig. 3

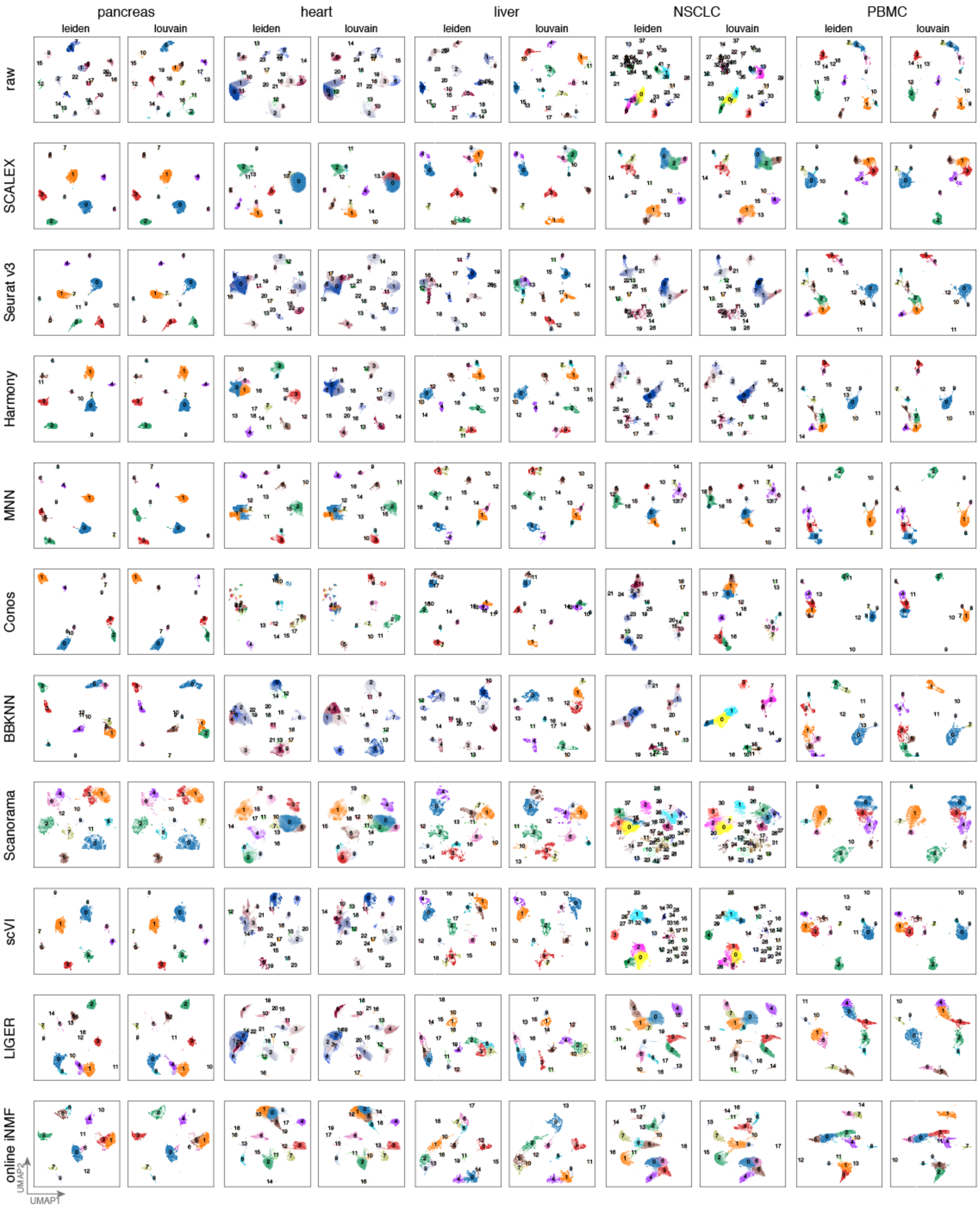

Supplementary Fig. 3 | Comparisons of clustering results of indicated methods across the indicated benchmark datasets. UMAP embeddings of clustering results of the indicated benchmark datasets after integration by indicated methods; colored by Leiden (left) and Louvain (right).

Supplementary Fig. 4

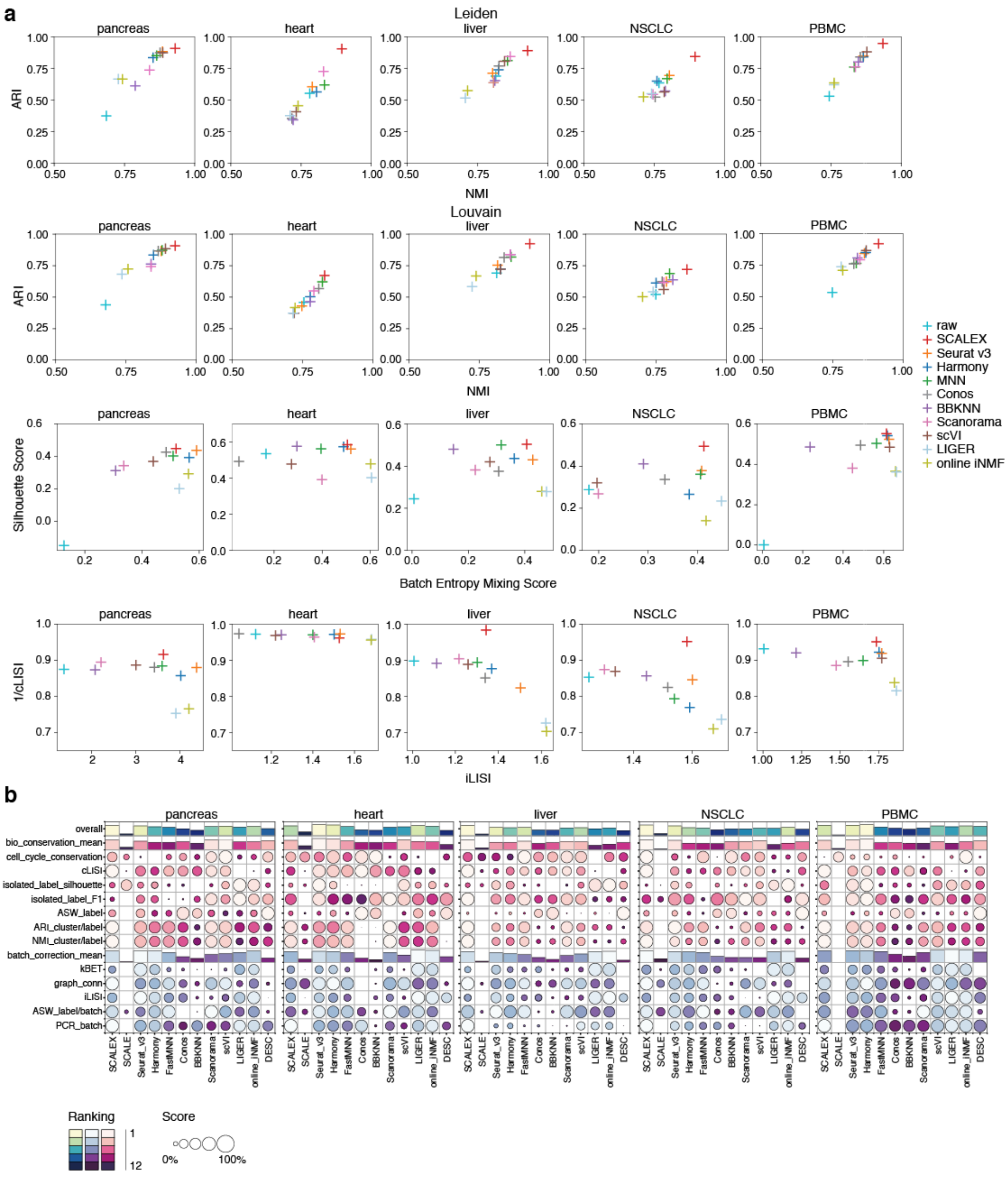

Supplementary Fig. 4 | Comparisons of integration performance by quantification metrics. **a**, Scatter plots showing the ARI and NMI scores based-on the Leiden and Louvain clustering results, the Silhouette and batch entropy mixing scores, and the cLISI/ iLISI scores. **b**, Dotplot showing the scores and rankings of indicated methods on a set of scIB metrics across the benchmark datasets. Note that we did not include the trajectory conservation score in comparison as it is designed for developmental analysis, nor the HVG conservation score because the calculations were failed for some methods.

Supplementary Fig. 5

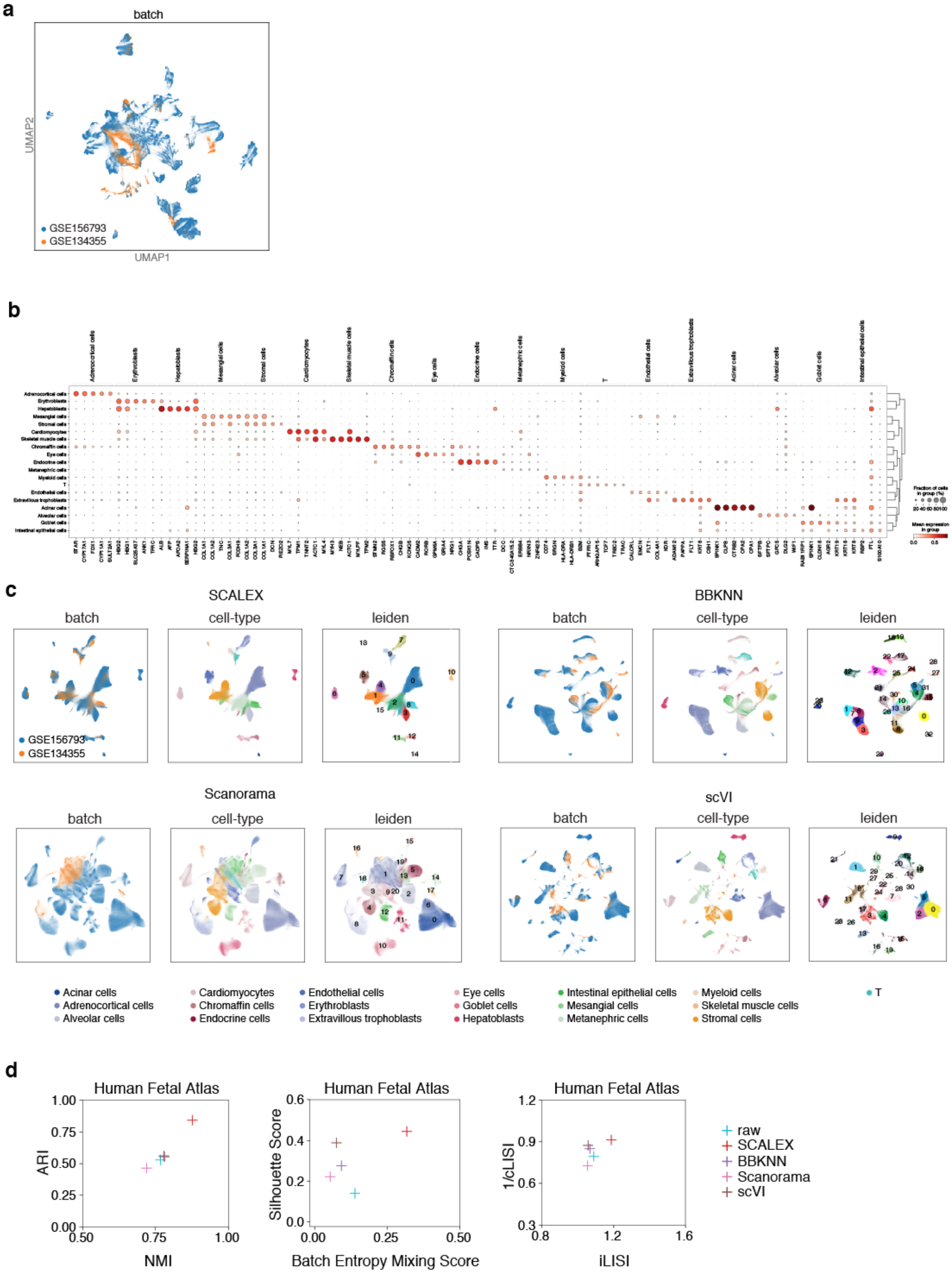

Supplementary Fig. 5 | Comparisons of integration performance of indicated methods based on Human Fetal Atlas dataset. a, UMAP embeddings of the Human Fetal Atlas before integration. **b,** Dotplot of canonical marker genes for each cell-type. Dot color represents average expression level, and dot size represents the proportion of cells in the group expressing the marker. **c**, UMAP embeddings of the indicated methods (only SCALEX, BBKNN, Scanorama, and scVI are scalable to the Human Fetal Atlas dataset). Cells are colored by batch (left), cell-type (middle), and Leiden clustering (right). **d**, Scatter plot showing the ARI and NMI scores based-on the Leiden clustering results, the Silhouette and batch entropy mixing scores, and the iLISI and 1/cLISI scores. Note that online iNMF was not successfully tested on the Human Fetal Atlas due to a HDF5 file conversion issue for large data.

Supplementary Fig. 6

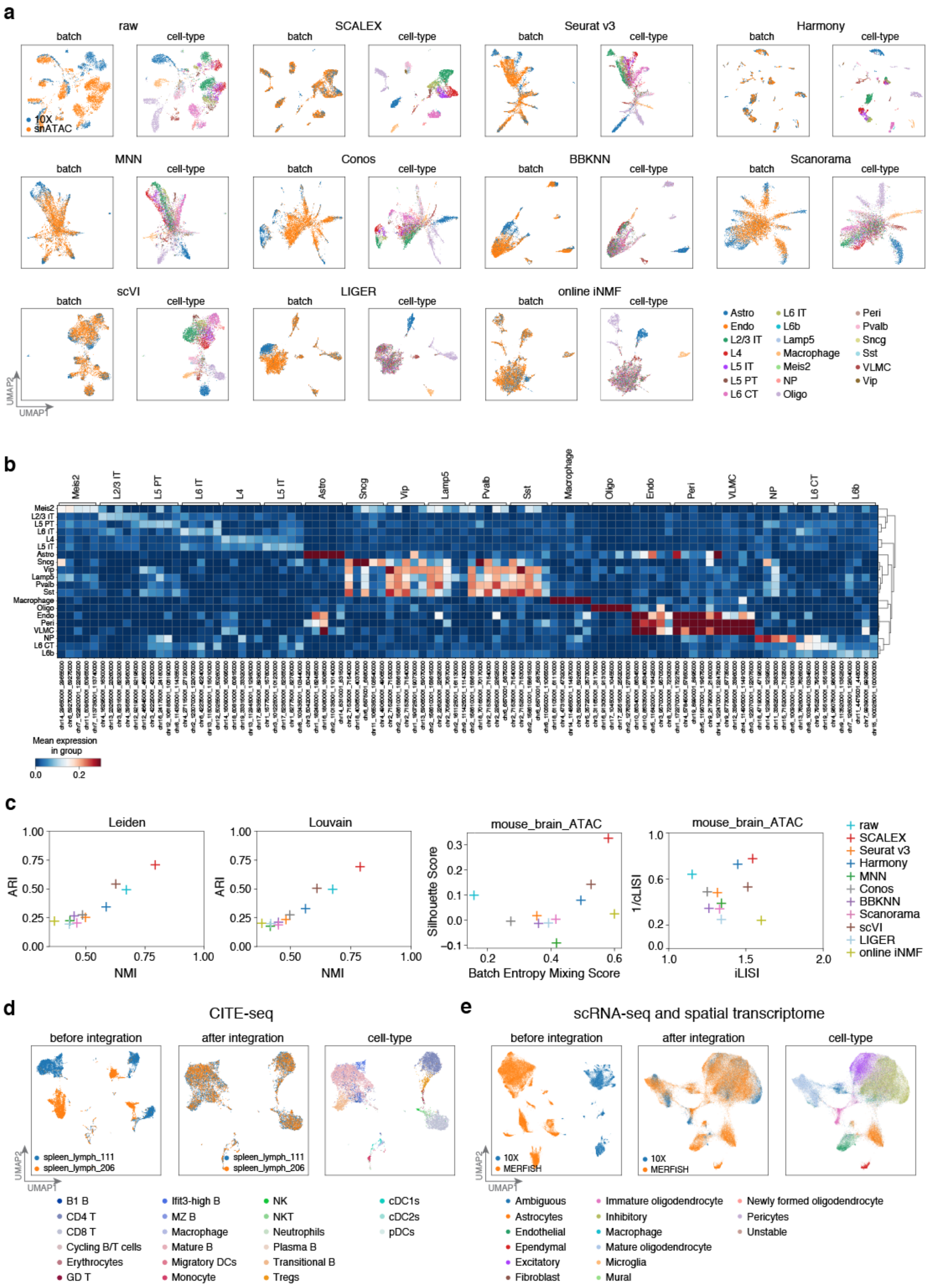

Supplementary Fig. 6 | Comparisons of integration performance of indicated methods based on scATAC-seq dataset and other modality datasets. a, UMAP embeddings of the mouse brain scATAC-seq dataset before and after integration by indicated methods; colored by batch (left) and cell-type (right). **b**, Cell-type-specific peaks for the mouse brain scATAC-seq dataset. **c**, Scatter plot showing the ARI and NMI scores based-on the Leiden and Louvain clustering results, the Silhouette and batch entropy mixing scores, and the iLISI and 1/cLISI scores. **d**, **e**, UMAP embedding before and after SCALEX integration over CITE-seq (which measures abundance of both RNA and protein in single cells) spleen lymph dataset and cross-modality mouse brain dataset between spatial transcriptome (MERFISH) dataset and RNA-seq (10X) dataset.

Supplementary Fig. 7

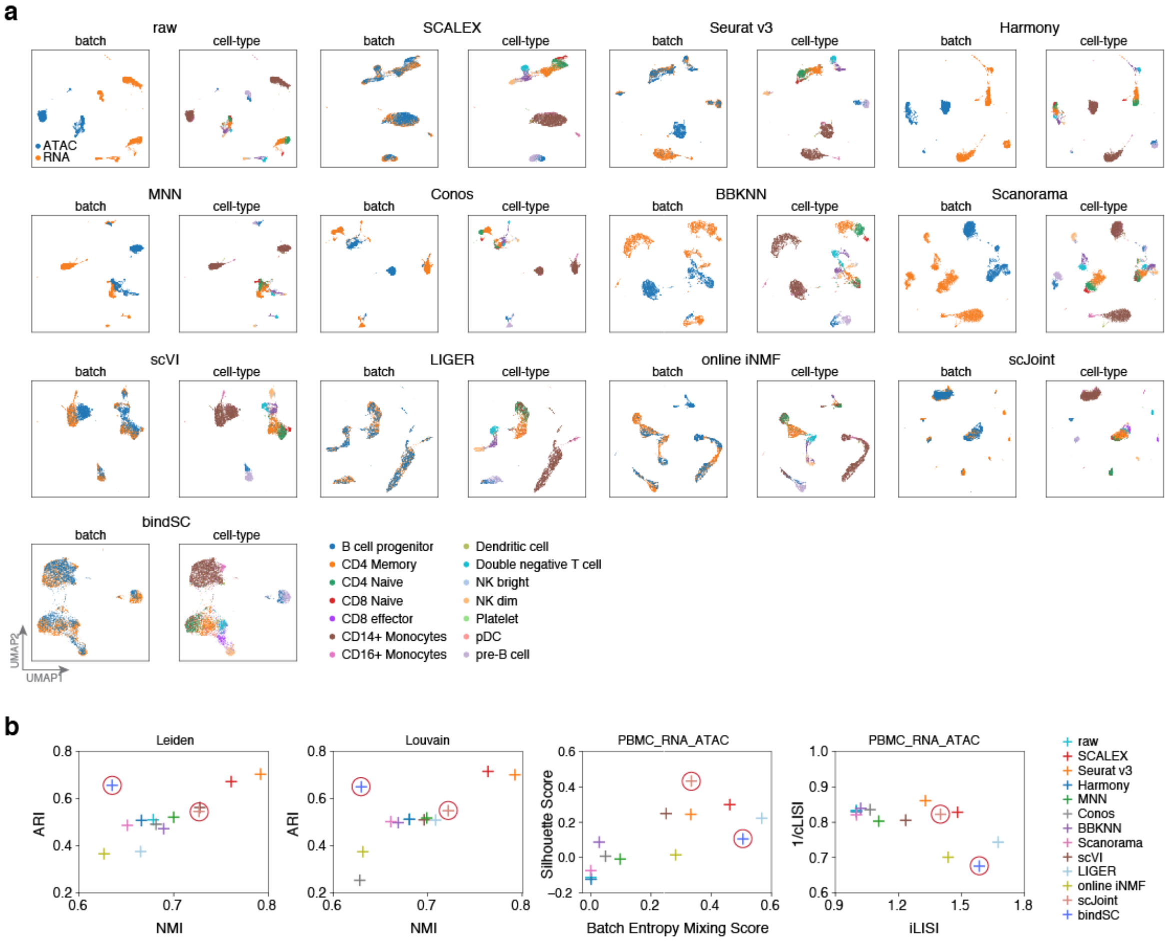

Supplementary Fig. 7 | Comparisons of integration performance of indicated methods based on cross-modality dataset. a, UMAP embeddings of the PBMC cross-modality dataset by indicated methods. Cells are colored by batch or cell-type. **b**, Scatter plot showing a quantitative comparison of the silhouette score (y-axis) and the batch entropy mixing score (x-axis) (left), ARI (y-axis), and NMI (x-axis) based-on Leiden clustering (middle) and Louvain clustering (right), the Silhouette and batch entropy mixing scores, and the iLISI and 1/cLISI scores for the PBMC cross-modality dataset. scJoint and bindSC are highlighted with red circles.

Supplementary Fig. 8

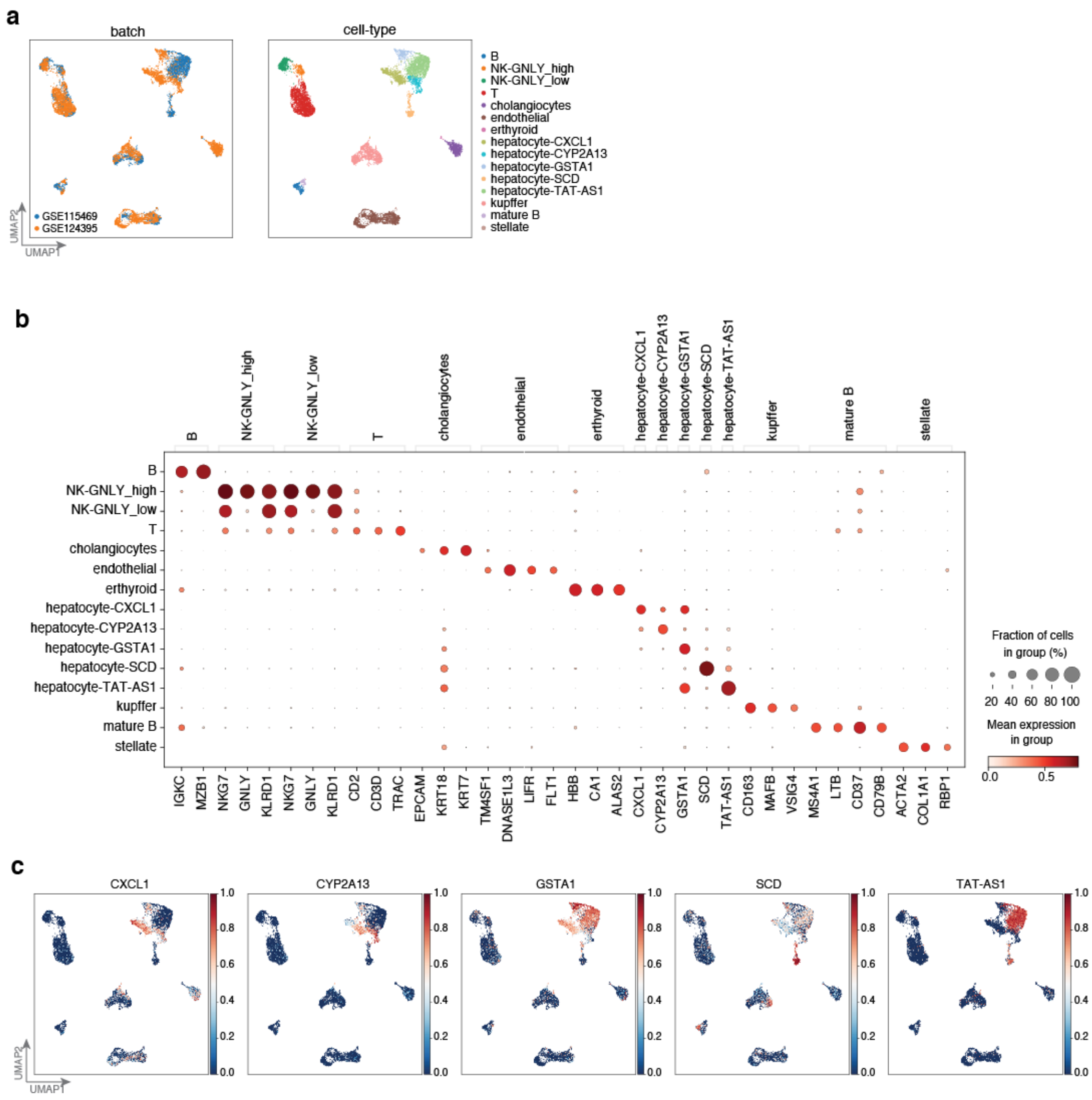

Supplementary Fig. 8 | Canonical marker genes of different cell-types and UMAP embeddings of the *liver* dataset. a, UMAP embeddings of the *liver* dataset, colored by batch (left) and cell-type (right) after SCALEX integration. **b,** Dotplot of canonical marker genes for each cell-type. Dot color represents average expression level, and dot size represents the proportion of cells in the group expressing the marker. **c**, Normalized marker gene expression on the UMAP embeddings of the five hepatocyte subtypes. Color bar represents the expression level.

Supplementary Fig. 9

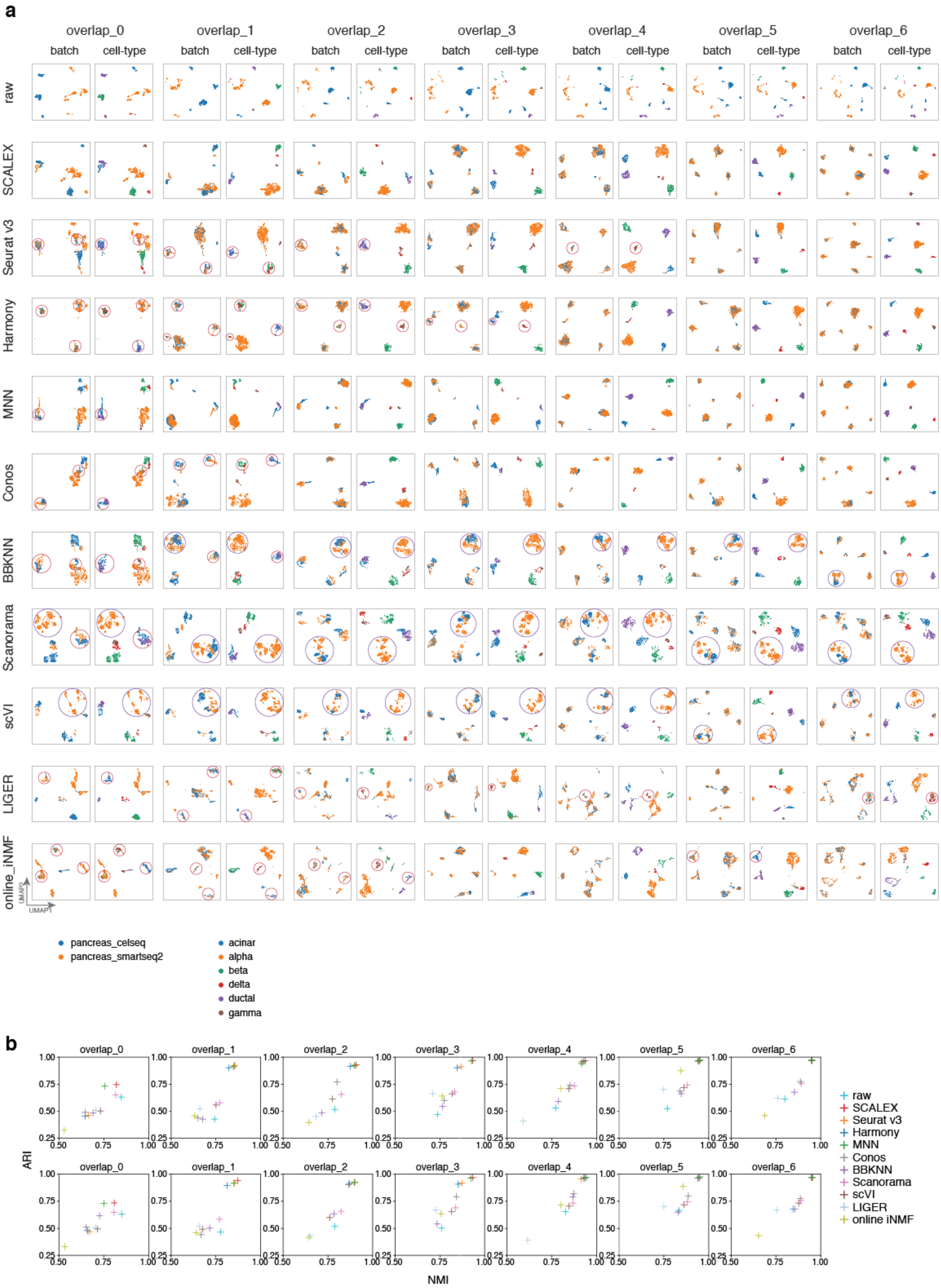

Supplementary Fig. 9 | Comparisons of integration performance based on partially overlapping simulated *pancreas* dataset. a, Partially overlapping datasets were generated by down-sampling the *pancreas* dataset, consisted of common cell-types with a decreased overlapping number (ranging from 0 to 6). Integration results for SCALEX, Seurat, Harmony, and online iNMF are shown in the UMAP embeddings colored by batches (left) and cell-types (right) respectively (overlapping number decreases from 6 to 0). Misalignments are highlighted with red circles. **b**, Scatter plot showing a quantitative comparison of the indicated methods in terms of the ARI score (y-axis) and the NMI score (x-axis), based on the Leiden (top) and Louvain (bottom) clustering results in the latent space based on simulated *pancreas* datasets.

Supplementary Fig. 10

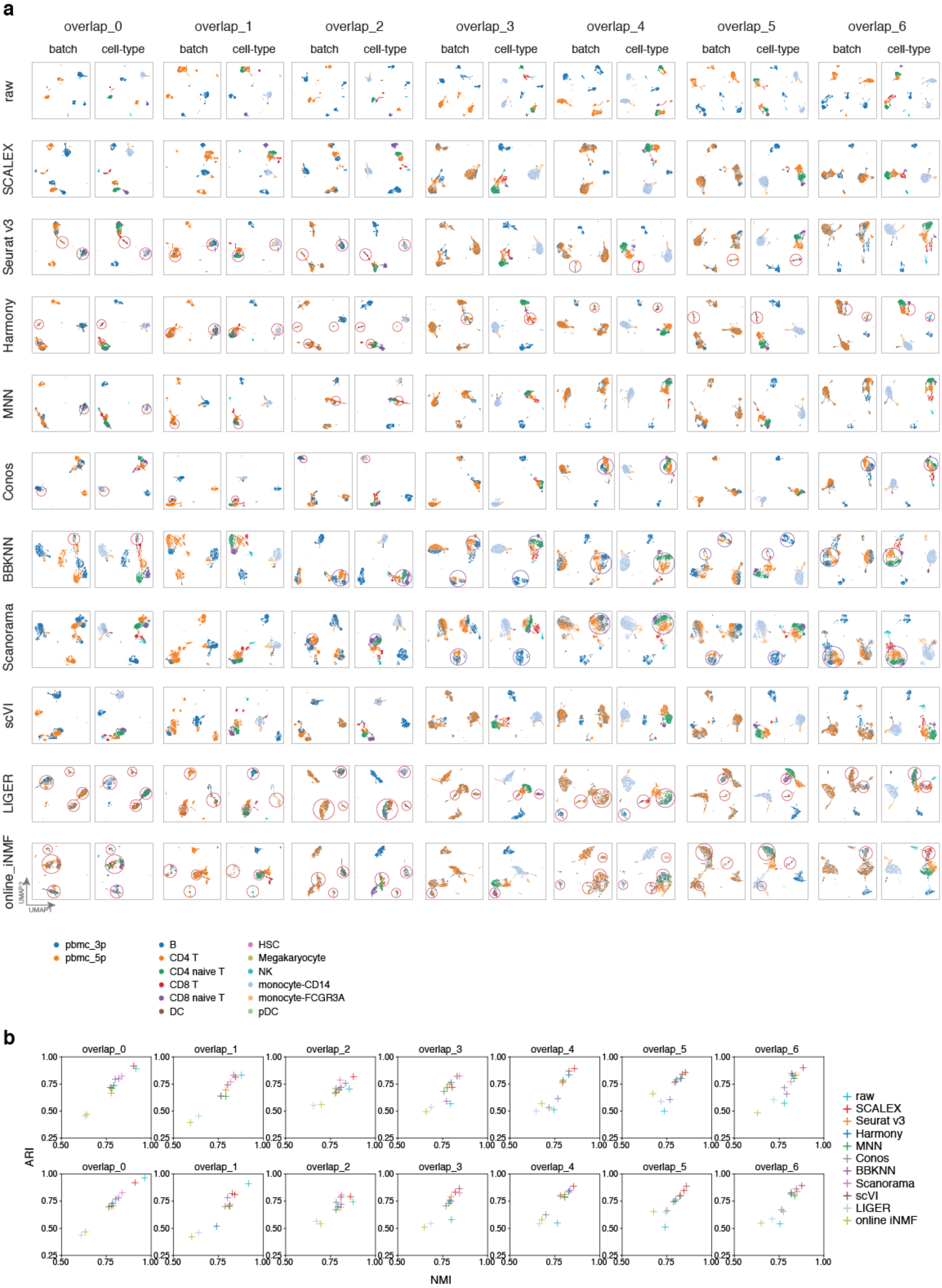

Supplementary Fig. 10 | Comparisons of integration performance based on partially overlapping simulated *PBMC* dataset. a, Partially overlapping datasets were generated by down-sampling the *PBMC* dataset, consisted of common cell-types with a decreased overlapping number (ranging from 0 to 6). Integration results for SCALEX, Seurat, Harmony, and online iNMF are shown in the UMAP embeddings colored by batches (left) and cell-types (right) respectively (overlapping number decreases from 6 to 0). Misalignments are highlighted with red circles. **b**, Scatter plot showing a quantitative comparison of the indicated methods in terms of the ARI score (y-axis) and the NMI score (x-axis), based on the Leiden (top) and Louvain (bottom) clustering results in the latent space based on simulated *PBMC* datasets.

Supplementary Fig. 11

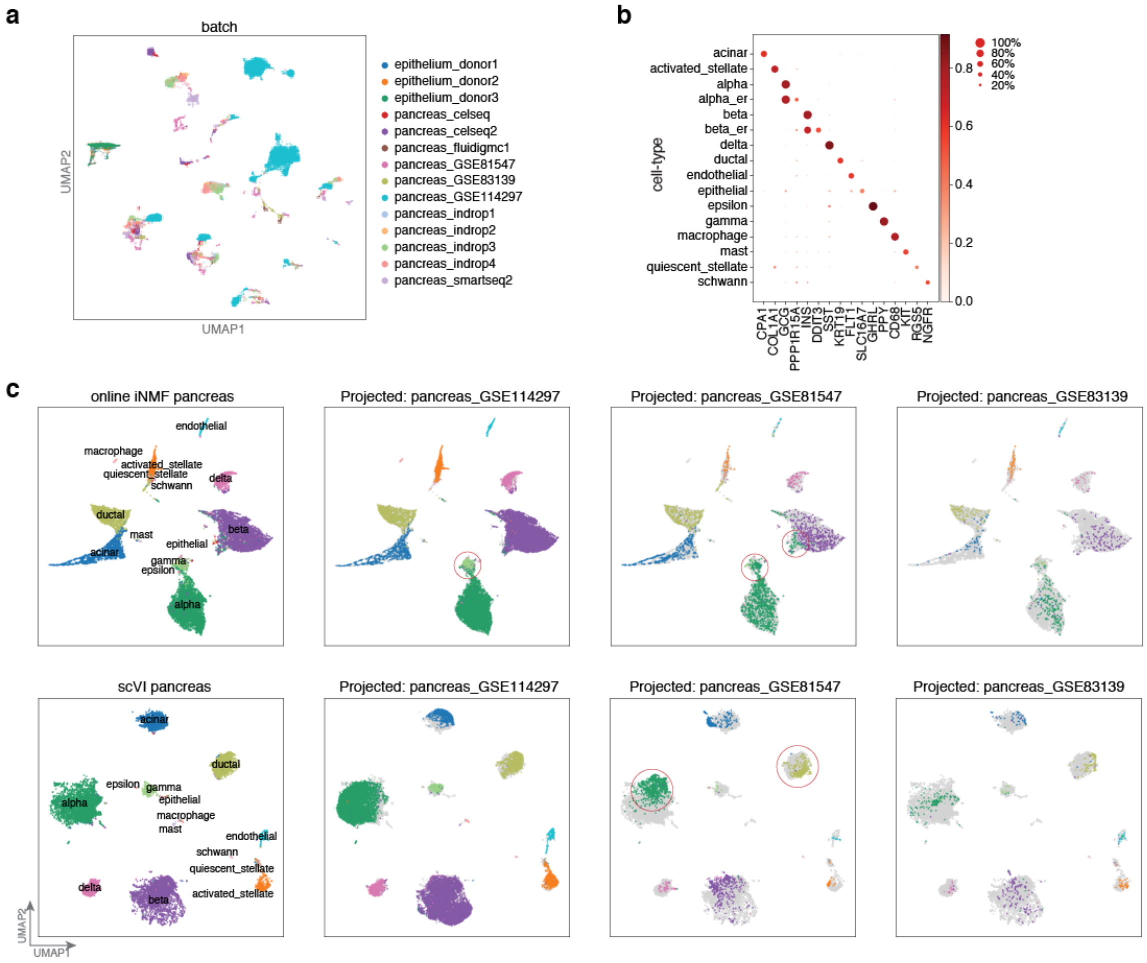

Supplementary Fig. 11 | Projection of three additional pancreas data batches onto the *pancreas* dataset. a, UMAP embeddings of the *pancreas* dataset and the three additional pancreas data batches and the bronchial epithelium data batches (data from three donors) before integration. Cells are colored by batch. **b**, Dot plot of canonical markers of cell-types of reference *pancreas* dataset; dot color represents average expression level, and dot size represents the proportion of cells in the group expressing the marker. **c,** UMAP embeddings of the common cell space obtained by using online iNMF (top) and scVI (bottom) to project the three additional indicated pancreas data batches onto the *pancreas* dataset. Cells are colored by cell-type with light gray shadows representing the original *pancreas* dataset. Misalignments are highlighted with red circles. Note that here for convenient comparison of the projected data with the existing data, we showed the UMAP embeddings of the *pancreas* dataset combined with the projected data, which are visualized

differently from the UMAP embeddings of the *pancreas* dataset alone in Supplementary Fig. 2 as UMAP visualizations change with embedding data.

Supplementary Fig. 12

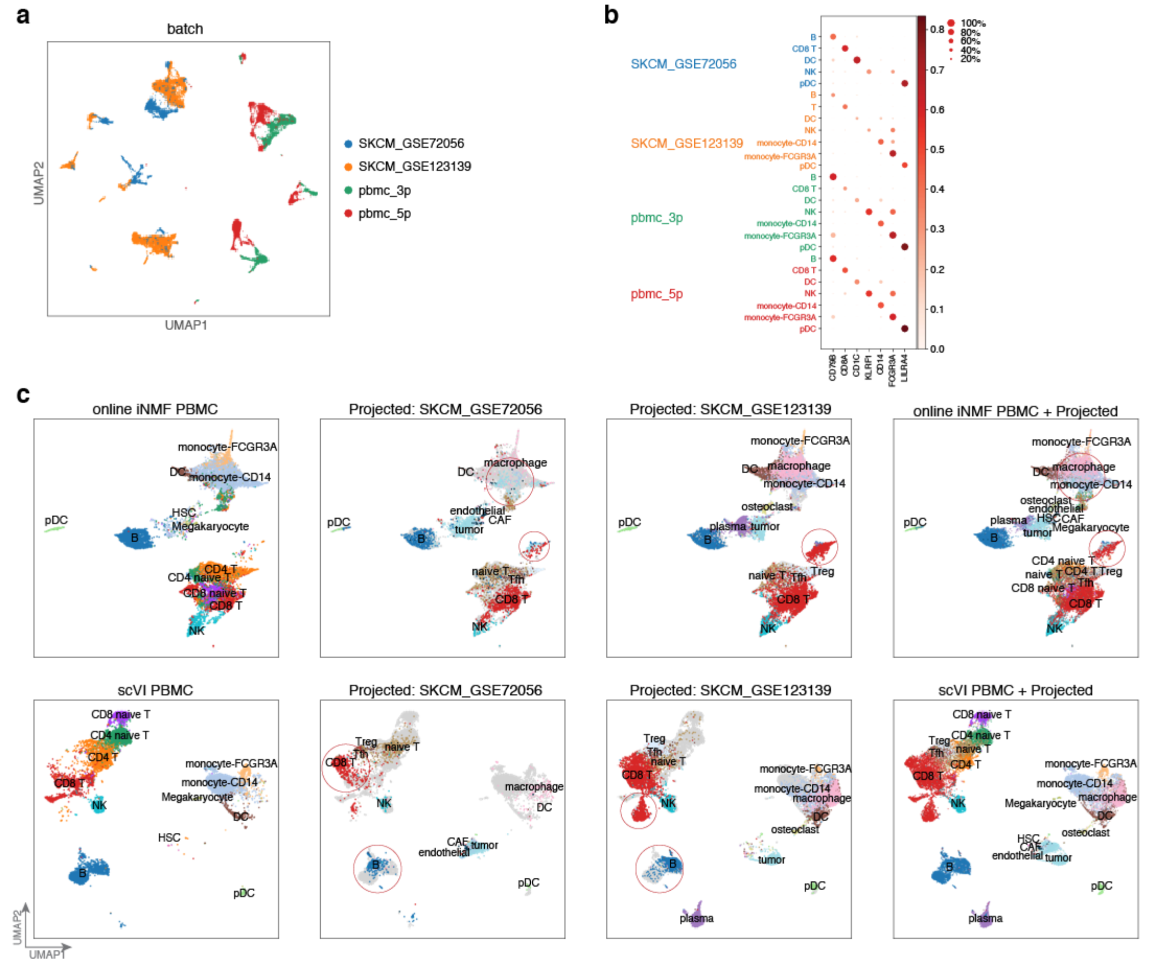

Supplementary Fig. 12 | Projection of two melanoma datasets onto the *PBMC* dataset. a, UMAP embeddings of the *PBMC* dataset and the two additional melanoma data batches before integration. Cells are colored by batch. **b**, Dot plot of canonical markers of cell-types of each batch; dot color represents average expression level, while dot size represents the proportion of cells in the group expressing the marker. **c**, UMAP embeddings of the common cell space obtained by using online iNMF (top) and scVI (bottom) to project the two additional indicated melanoma data batches onto the *PBMC* dataset. Cells are colored by cell-type with light gray shadows representing the original *PBMC* dataset. Misalignments are highlighted with red circles. Note that here for convenient comparison of the projected data with the existing data, we showed the UMAP embeddings of the *PBMC* dataset combined with the projected data, which are visualized

differently from the UMAP embeddings of the *PBMC* dataset alone in Supplementary Fig. 2 as UMAP visualizations change with embedding data.

Supplementary Fig. 13

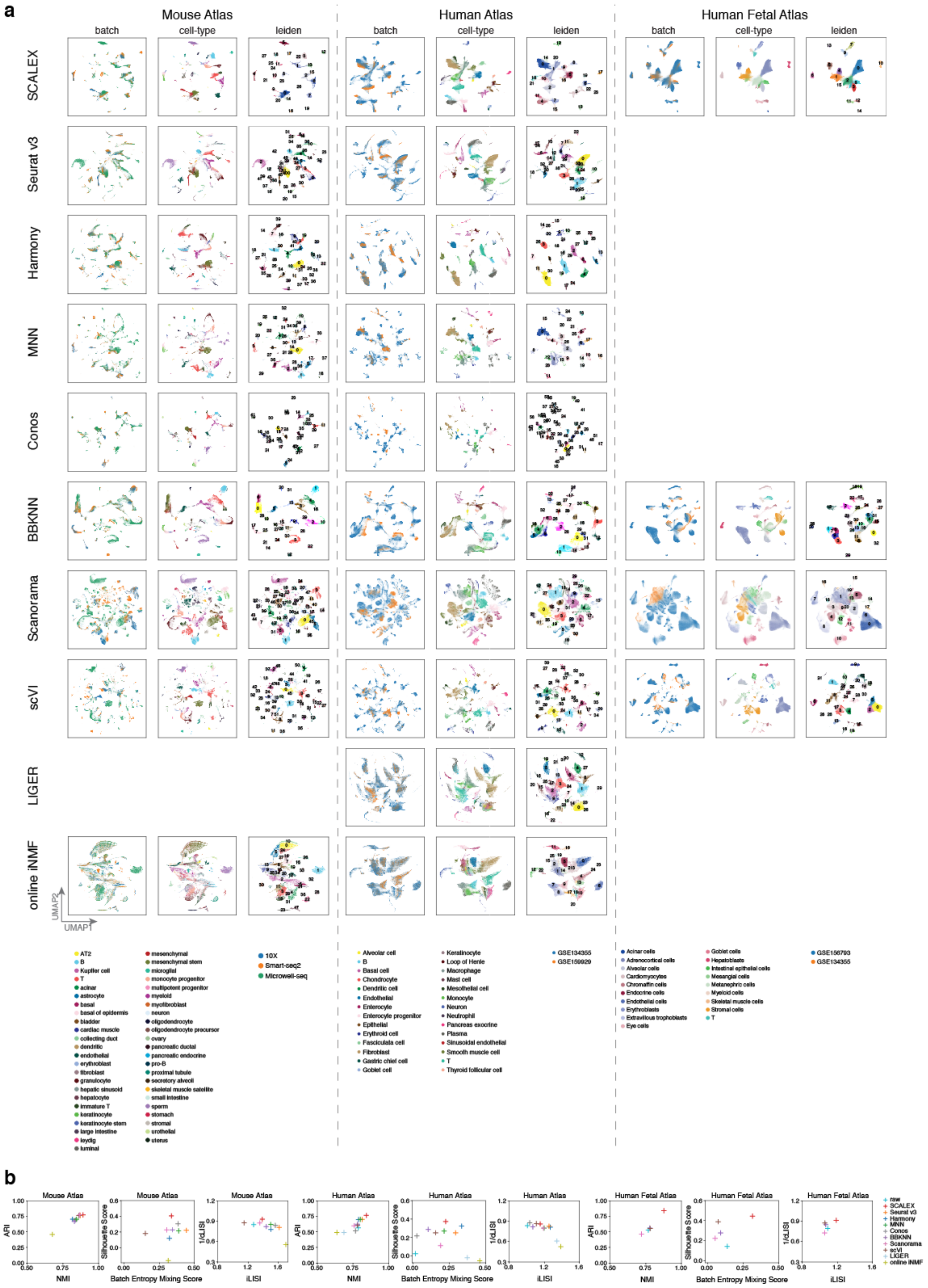

Supplementary Fig. 13 | Comparisons of integration across Atlas-level datasets. a, UMAP embeddings of the indicated methods across the indicated datasets. Results are blank for those methods not scalable to this data size. **b**, Scatter plots showing the comparisons of indicated methods in terms of the ARI and NMI scores based-on the Louvain clustering results, the Silhouette and batch entropy mixing scores, and the cLISI/ iLISI scores based on the indicated Atlas-level datasets.

Supplementary Fig. 14

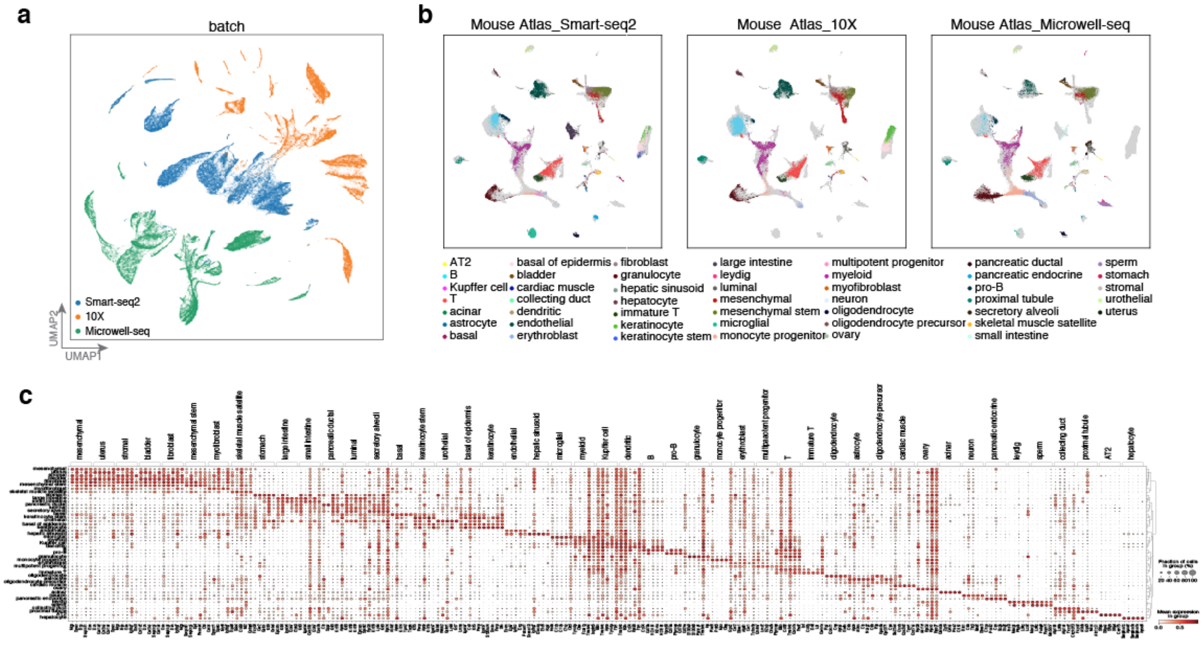

Supplementary Fig. 14 | The SCALEX Mouse Atlas. a, UMAP embeddings of the Mouse Atlas dataset before integration, colored by batch. **b**, UMAP embeddings of three mouse atlas data batches (Smart-seq2, 10X, and Microwell-seq) after integration, colored by cell-type; the light gray shadows represent the original Mouse Atlas dataset. **c**, Dotplot of the top 5 cell-type-specific genes for each cell-type in the Mouse Atlas dataset. Dot color represents average expression level, while dot size represents the proportion of cells in the group expressing the marker. Note that here for convenient comparison of the projected data with the existing data, we showed the UMAP embeddings of the Mouse Atlas combined with the projected data, which are visualized differently from the UMAP embeddings of the Mouse Atlas alone in Supplementary Fig. 13 as UMAP visualizations change with embedding data.

Supplementary Fig. 15

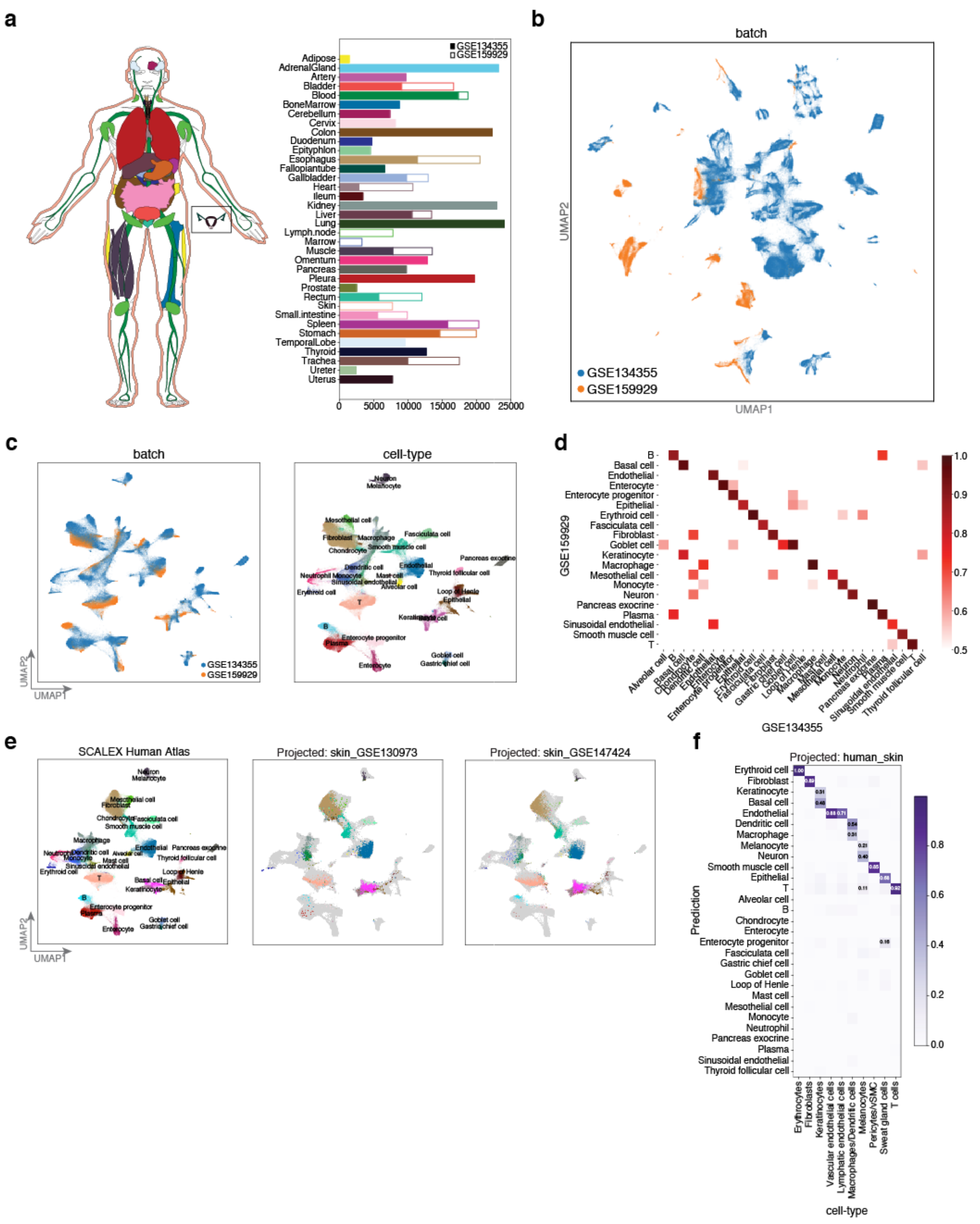

Supplementary Fig. 15 | The SCALEX Human Atlas. a, The Human Atlas dataset acquired using different technologies (Smart-seq2, 10X, and Microwell-seq) covering various tissues used for construction of the human atlas. **b-c**, UMAP embeddings of the Human Atlas dataset colored by batch and cell-type, before (**b**) and after integration (**c**). **d**, Similarity matrix of meta-cell

representations for cell-types in the two data batches in the common cell-embedding space after SCALEX integration between two batches. Color bar represents the Pearson correlation coefficient between the average meta-cell representation of two cell-types from a respective data batch. **e**, UMAP embeddings of the common cell space obtained by using SCALEX to project the two additional indicated human skin data batches onto the Human Atlas dataset. Cells are colored by cell-type with light gray shadows representing the original Human Atlas dataset. **f**, Confusion matrix of the cell-type annotations by SCALEX and those in the original study. Color bar represents the percentage of cells in confusion matrix C_{ij} known to be in cell-type i and predicted to be in cell-type j. Note that here for convenient comparison of the projected data with the existing data, we showed the UMAP embeddings of the Human Atlas combined with the projected data, which are visualized differently from the UMAP embeddings of Human Atlas alone in Supplementary Fig. 13 as UMAP visualizations change with embedding data.

Supplementary Fig. 16

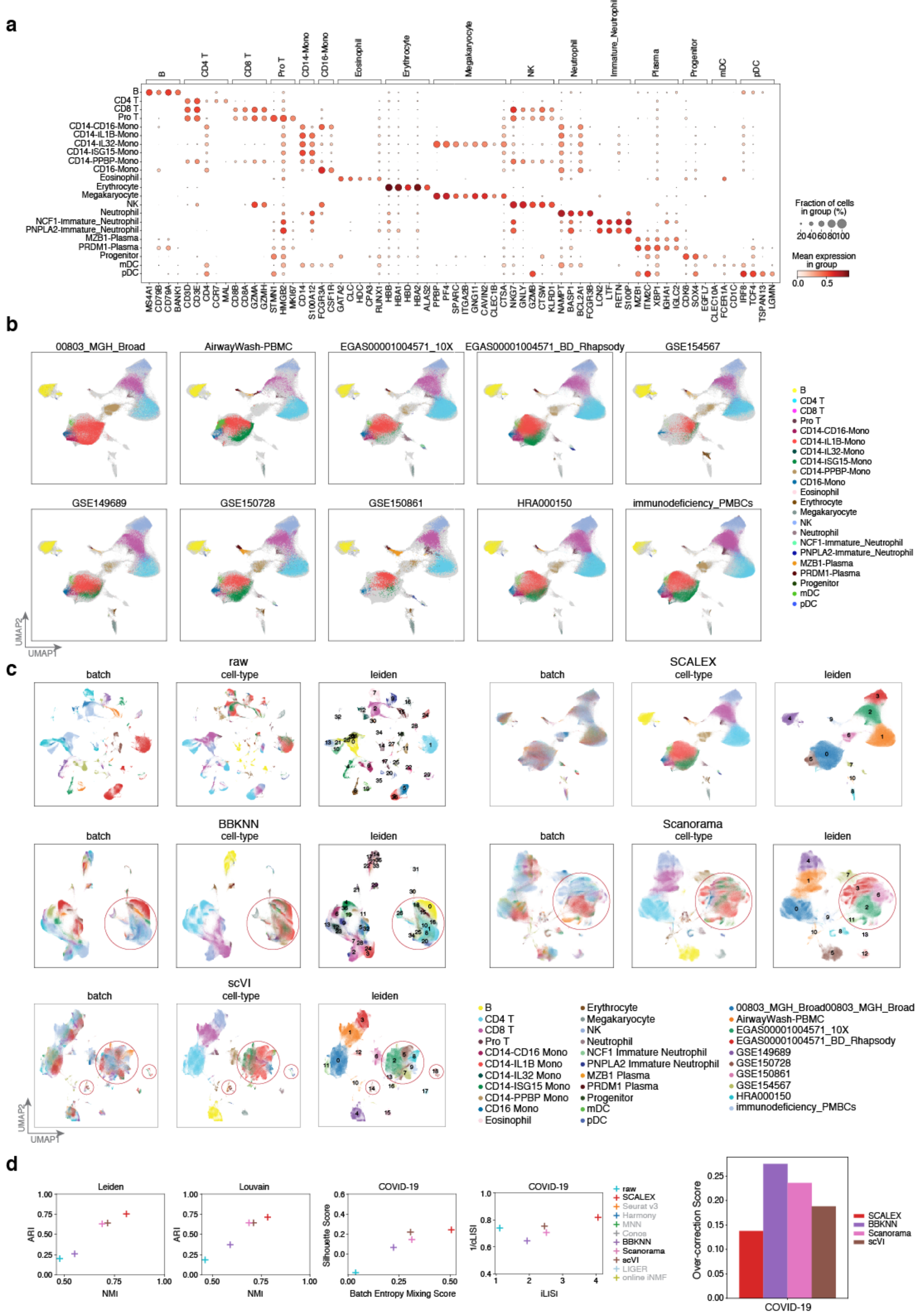

Supplementary Fig. 16 | COVID-19 immune landscape. a, Dotplot of canonical marker genes for each cell-type. Dot color represents average expression level, while dot size represents the proportion of cells in the group expressing the marker. **b**, UMAP embeddings of the COVID-19 PBMC atlas in individual batches after SCALEX integration, colored by cell-type; the light gray shadows represent the other batches of COVID-19 PBMC atlas. **c**, UMAP embeddings of the COVID-19 PBMC Atlas dataset before and after integration by indicated methods. Cells are colored by batch (left), cell-type (middle) and Leiden clustering results (right). **d**, Scatter plots showing the comparisons of indicated methods in terms of the ARI and NMI scores based-on the Leiden and Louvain clustering results, the Silhouette and batch entropy mixing scores, and the cLISI/ iLISI scores. Bar plot showing the comparisons of indicated methods in terms of the over-correction scores based on the COVID-19 PBMC Atlas dataset.

Supplementary Fig. 17

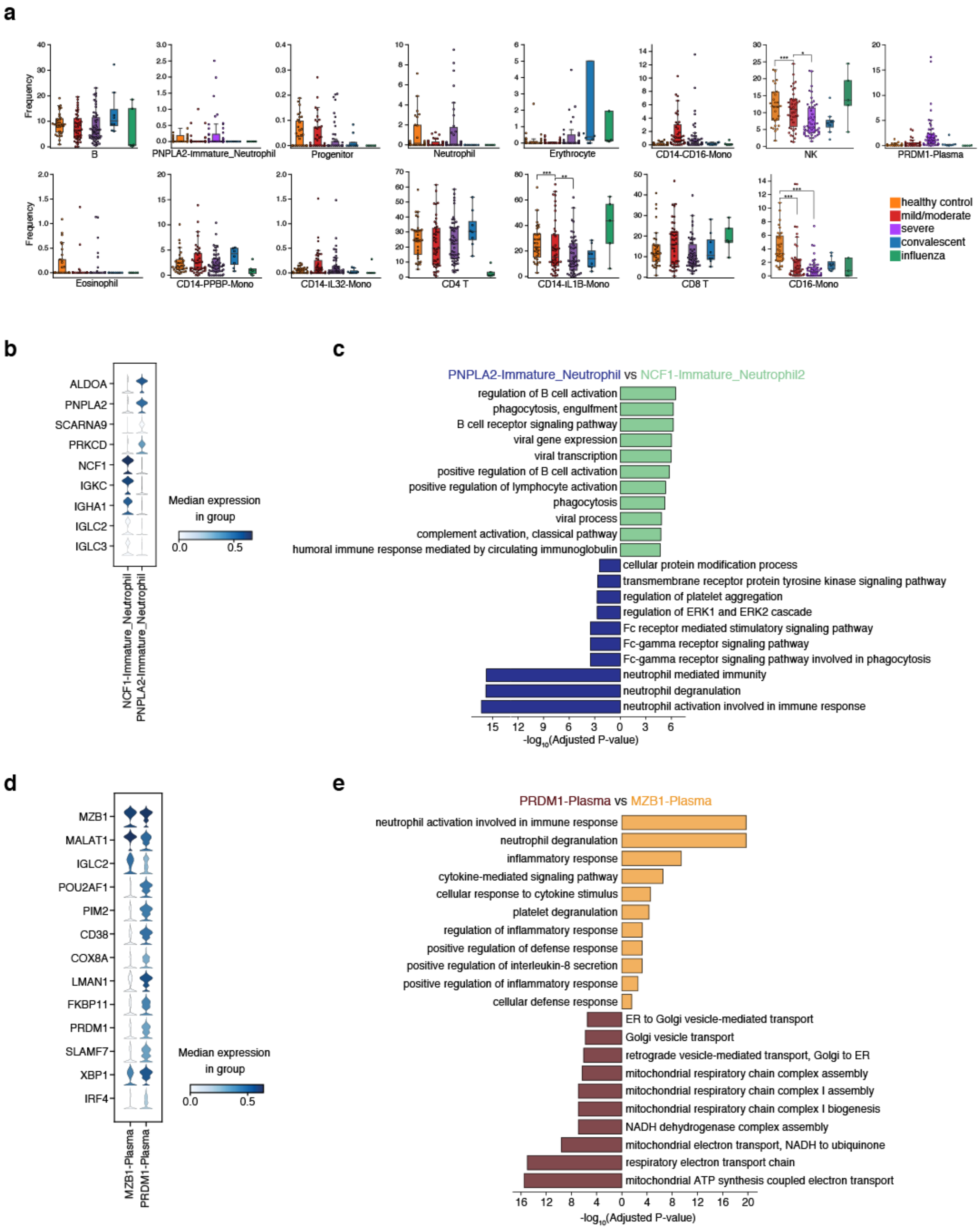

Supplementary Fig. 17 | COVID-19 heterogeneous dysfunctional immune response. a, Frequency of cell distributions across healthy people (n=31) and influenza patient controls (n=5), and among mild/moderate (n=46), severe (n=50), and convalescent (n=12) COVID-19 patients.

Dirichlet-multinomial regression was used for pairwise comparisons, two-sided *t*-test, NK, healthy control vs mild/moderate: p=2.78×10^{-8}, mild/moderate vs severe: p=0.015; CD14-IL1B-Mono, healthy control vs mild/moderate: p=2.34×10^{-17}, mild/moderate vs severe: p=0.0039; CD16-Mono, healthy control vs mild/moderate: p=1.58×10^{-11}, healthy control vs severe: p=1.08×10^{-16}. ***p<0.001, **p<0.01, *p<0.05. Midline, median; boxes, interquartile range; whiskers, 1.5× interquartile range. **b**, Stacked violin plot of differentially-expressed genes between PNPLA2-Immature_Neutrophil and NCF1-Immature_Neutrophil cells. **c**, GO terms enriched in the differentially-expressed genes for PNPLA2-Immature_Neutrophil and NCF1-Immature_Neutrophil cells. Hypergeometric test, *p* values were adjusted using the Benjamini-Hochberg method. **d**, Stacked violin plot of differentially-expressed genes between PRDM1-Plasma and MZB1-Plasma. **e**, GO terms enriched in the differentially-expressed genes for PRDM1-Plasma and MZB1-Plasma cells. Hypergeometric test, *p* values were adjusted using the Benjamini-Hochberg method.

Supplementary Fig. 18

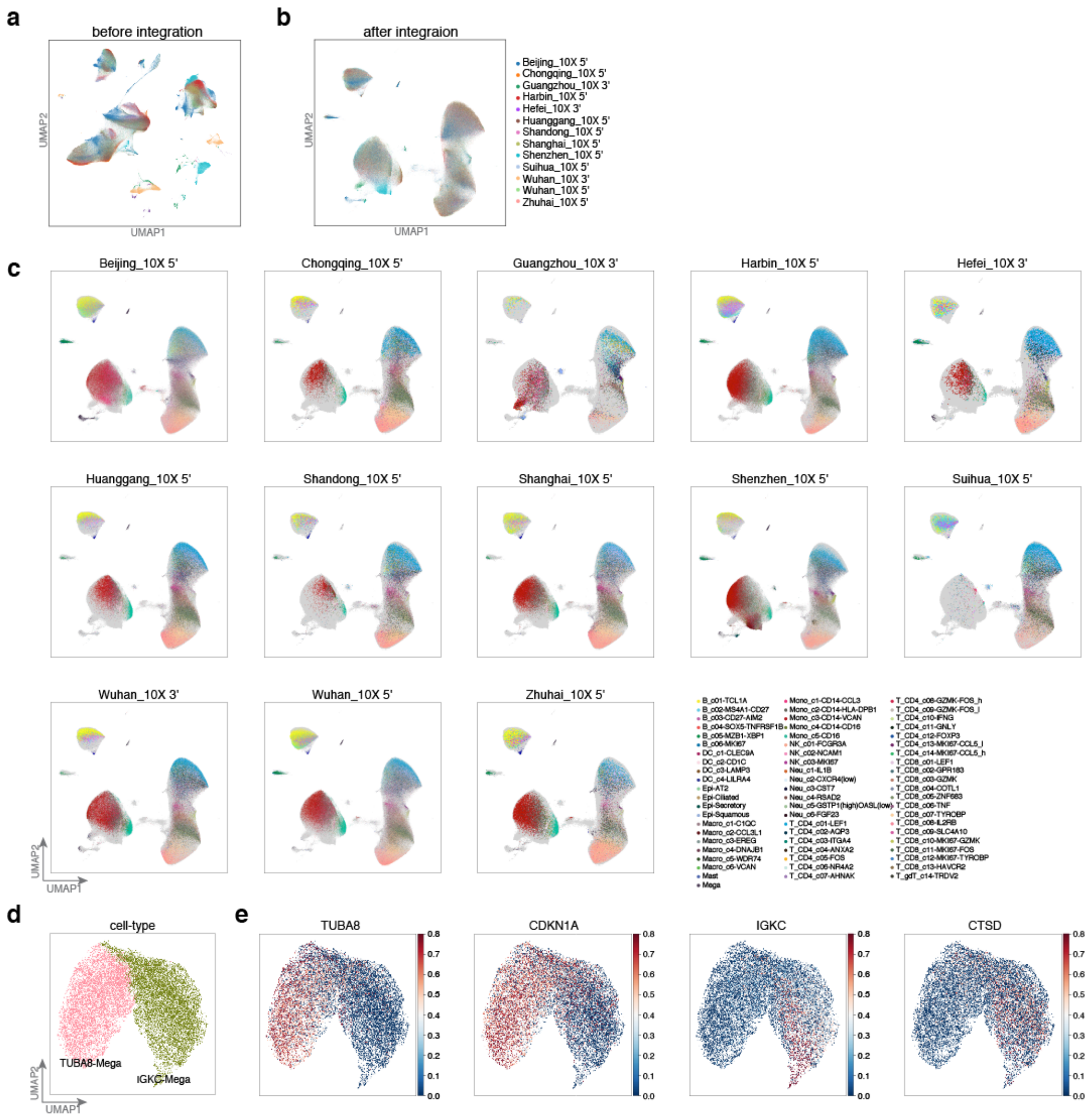

Supplementary Fig. 18 | Projection of the SC4 Atlas onto the SCALEX COVID-19 PBMC Atlas. a-b, UMAP embeddings of the SC4 Atlas before integration (**a**) and after projection onto the SCALEX COVID-19 PBMC Atlas (**b**). **c**, Separate UMAP embeddings of each SC4 data batch, after being projected onto the SCALEX COVID-19 PBMC space, colored by cell-type. light gray shadows represent the COVID-19 PBMC Atlas. **d**, UMAP embeddings of the TUBA8-Mega and IGKC-Mega cells. **e**, UMAP embeddings of the differentially-expressed genes of TUBA8-Mega and IGKC-Mega cells.

Supplementary Fig. 19

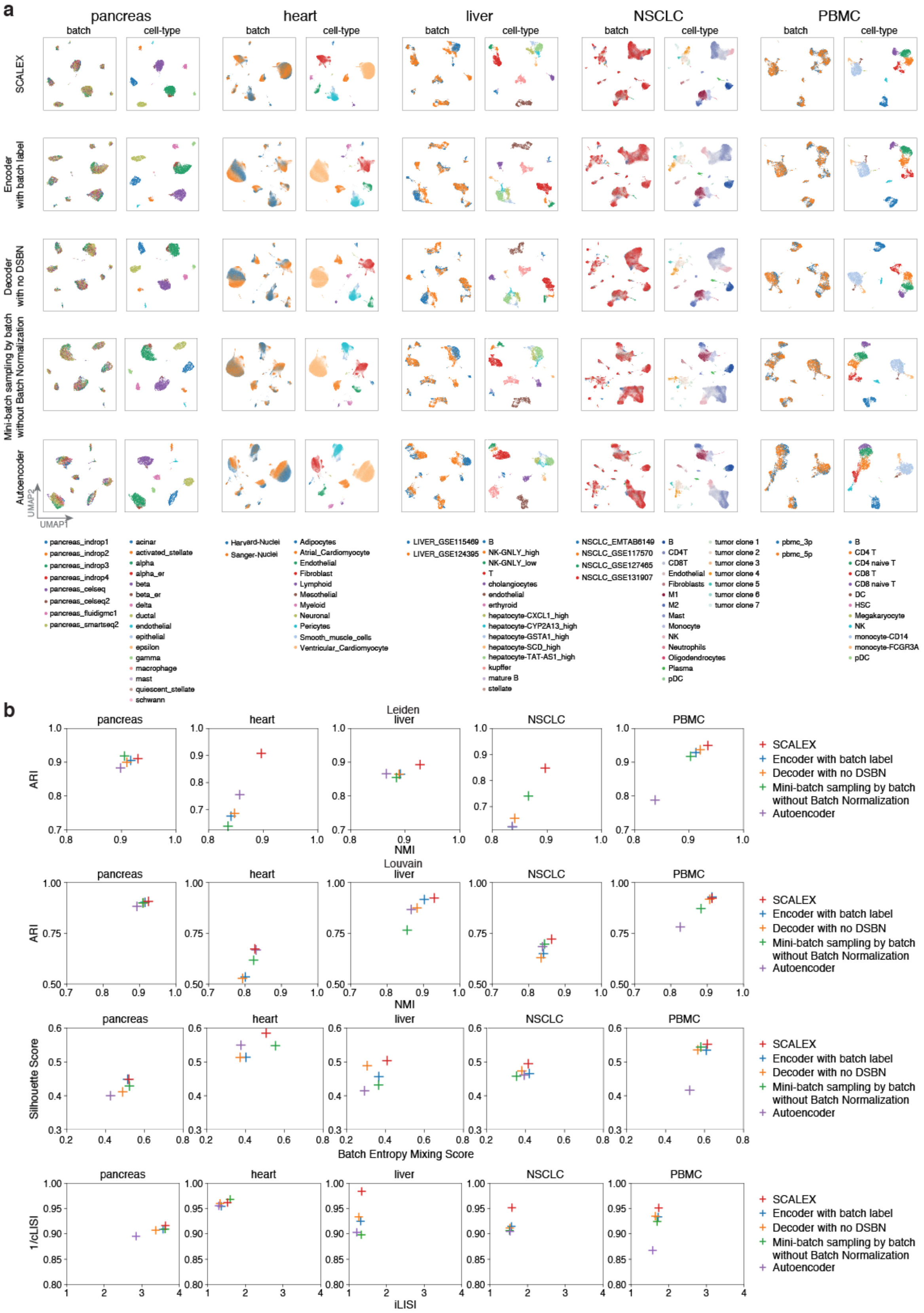

Supplementary Fig. 19 | Ablation studies of different SCALEX architectures for single-cell data integration. a, UMAP embeddings after integration by SCALEX and other SCALEX test-variants. **b**, Scatter plot showing a quantitative comparison of the performance of the full and different test-variants of SCALEX using the ARI score (y-axis) and the NMI score (x-axis), the Silhouette score (y-axis) and the batch entropy mixing score (x-axis), and the 1/cLISI score (y-axis) and the iLISI score (x-axis), across the indicated benchmark datasets.

Supplementary Fig. 20

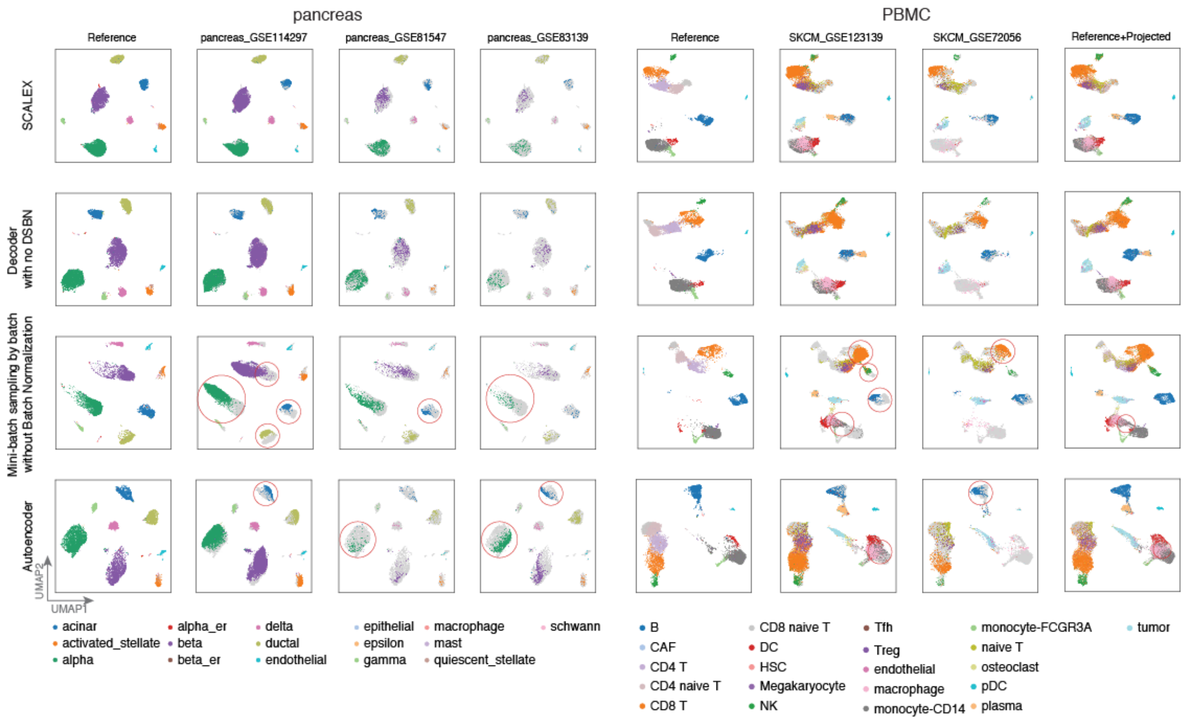

Supplementary Fig. 20 | Ablations studies of different SCALEX architectures for single-cell data projection. Left: UMAP embeddings of three projected pancreas data batches projected onto the pancreas space using different SCALEX architectures, colored by cell-type; light gray shadows represent the original *pancreas* dataset. Right: UMAP embeddings of the two projected melanoma data batches projected onto the PBMC space using different SCALEX architectures, colored by cell-type; light gray shadows represent the original *PBMC* dataset.

Supplementary Fig. 21

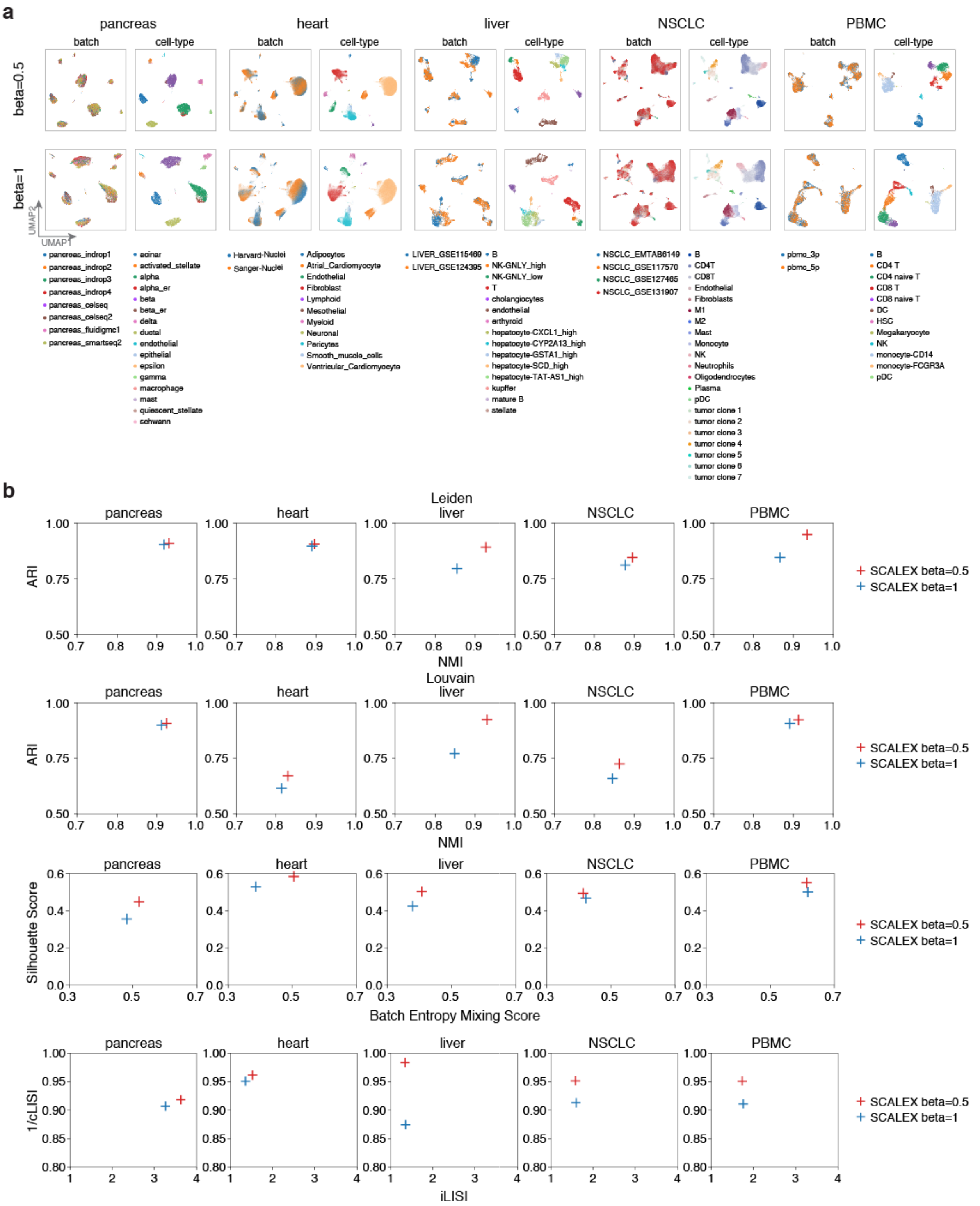

Supplementary Fig. 21 | Comparison of SCALEX with beta=0.5 and beta=1 across the indicated benchmark datasets. a, UMAP embeddings of SCALEX (beta=0.5) and SCALEX

(beta=1) across the indicated benchmark datasets; colored by batch (left) and cell-type (right). **b**, Scatter plot showing a quantitative comparison of the performance of SCALEX (beta=0.5) and SCALEX (beta=1) using the ARI score (y-axis) and the NMI score (x-axis), the Silhouette score (y-axis) and the batch entropy mixing score (x-axis), and the 1/cLISI score (y-axis) and the iLISI score (x-axis), across the indicated benchmark datasets.
