# Supplementary material for: Online single-cell data integration through projecting heterogeneous datasets into a common cell-embedding space
Source: Nat Commun. 2022 Oct 17;13:6118. doi: 10.1038/s41467-022-33758-z (PMC9574176; doi:10.1038/s41467-022-33758-z)
Supplement: Supplementary file 3 — Description for Additional Supplementary Files [file 41467_2022_33758_MOESM3_ESM.pdf]

Description of Additional Supplementary Information Files Document

Supplementary Dataset 1 l datasets.xlsx – The details of the public datasets

Supplementary Dataset 2 l clustering information.xlsx - Parameters and cluster numbers of different Methods

Supplementary Dataset 3 l Inflammatory and cytokine genes.xlsx - Inflammatory and cytokine genes
